# THE *‘HOLI’* DERMATOSES: ANNUAL SPATE OF SKIN DISEASES FOLLOWING THE SPRING FESTIVAL IN INDIA

**DOI:** 10.4103/0019-5154.55632

**Published:** 2009

**Authors:** Sudip Kumar Ghosh, Debabrata Bandyopadhyay, Gobinda Chatterjee, Debabrata Saha

**Affiliations:** *From the Departments of Dermatology, Venereology, and Leprosy, R.G. Kar Medical College, Kolkata, India.*

**Keywords:** *Cultural*, *dermatoses*, *Holi*, *India*, *spring festival*

## Abstract

**Background:**

‘Holi’ is an annual spring festival celebrated all over India. The central ritual of Holi involves throwing of colors on one another. Playing with toxic industrial dyes often results in various dermatological complaints in a significant number of people immediately following the celebration.

**Aims:**

To describe patterns of various skin manifestations directly or indirectly related to the use of different colors in the celebration of Holi.

**Methods:**

Observational clinical study on consecutive patients presenting to a teaching hospital in Kolkata, India.

**Results:**

Forty-two patients with a mean age of 24.2 years were studied. Itching was the commonest symptom (25, 59.5%), followed by burning sensation, pain, oozing, and scaling. Eleven patients’ symptoms were attributed to activities related to preparation of colors and the removal of colors from the skin surface. Eczematous lesions were the most common pattern (24, 57.1%) followed by erosions, xerosis and scaling, erythema, urticaria, acute nail-fold inflammation, and abrasions. Thirteen (30.9%) patients reported aggravation of preexisting dermatoses (acne, eczema, and paronychia). Secondary pyoderma occurred in 3 (7.1%). Face was the commonest site affected (24, 57.1%), followed by dorsum of the hands, scalp, forearm, palms, arms, and trunk. Ocular complaints in the form of redness, watering, and grittiness occurred in 7 (16.7%) patients.

**Conclusion:**

Various forms of cutaneous manifestations, often associated with ocular complaints, occur commonly due to Holi colors. Public awareness and regulatory actions are needed to avoid these preventable conditions.

## Introduction

Cultural and religious practices may have significant impact on the health of people. Some practices may often introduce health hazards, including skin diseases. One such cultural and religious practice is *Holi*, an annual festival celebrated all over India since ancient times. Holi begins on the day after the full moon in the Hindu month of *Phalguna* (February-March). Originally, an agricultural festival of fertility and harvest to welcome the spring, *Holi* also commemorates some Hindu mythology and legends. *Holi* is one of the most popular annual events in India, observed with great festivity and hilarity. In the eastern Indian state of West Bengal and in the surroundings of the city of Kolkata, it is popularly known as *Doljatra* or *Basonto- Utsav* (‘spring festival’). On the day of *Holi*, people gather together in a common place where they traditionally celebrate by applying colors in different forms on friends, family, and even complete strangers. Originally, the bright flowers that blossomed during spring acted as raw materials from which the different shades of *Holi* colors were made. Most of these trees were supposed to have medicinal properties beneficial to the skin. Over the years, with the spread of industrialization and urbanization, natural colors came to be replaced by inexpensive industrial dyes manufactured through chemical processes. A large number of patients consult dermatologists over the few days immediately following *Holi* for skin problems arising out of celebration with these colors. Although widely recognized and discussed as an annually recurring problem involving many people, lack of any formal study on it has prompted us to undertake the present work. We intend to describe the patterns of cutaneous manifestations in patients resulting from the colors and associated complications.

## Materials and Methods

Consecutive patients presenting with dermatological problems directly or indirectly attributed to the *Holi* colors to the Dermatology Clinic of a teaching hospital at Kolkata, India, were included in this observational clinical study. This year (2008), the *Holi* was observed on 21^st^ March. The study duration included the next two weeks, because this was the most probable time of presentation for *Holi*-related dermatoses. Detailed history regarding age, gender, symptoms, type of color used, activities related to preparation and removal of colors from the skin, and history of aggravation of preexisting dermatoses were recorded. Every patient then underwent a thorough clinical examination with special reference to the morphology of the lesions and sites of involvement.

## Result

**Figure 1:**
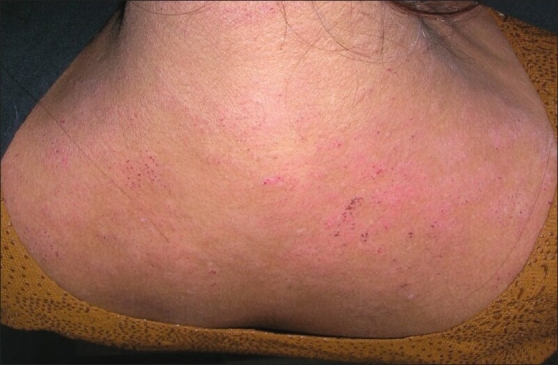
Contact dermatitis on back due to red color

**Figure 2:**
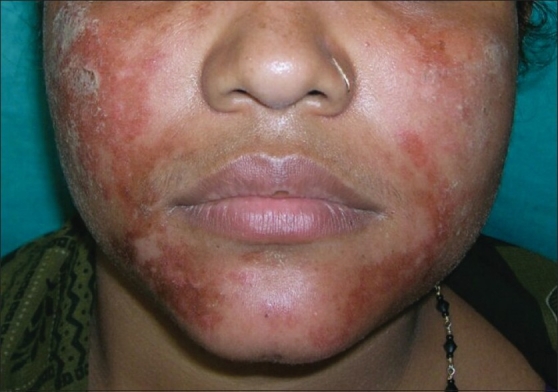
Facial erosive lesions due to color and vigorous rubbing

**Figure 3:**
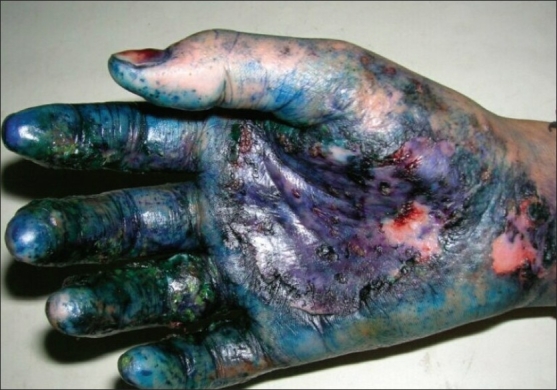
Acute erosive and crusted lesions following handling of blue and green colors

A total of 42 patients were evaluated. All the patients were residents of the state of West Bengal comprised by both urban population of the city of Kolkata and its surrounding rural districts. Their ages ranged from 4 to 46 years with a mean of 24.2. One third of the patients (14) belonged to the 20–30 years age group. There was a slight female preponderance (F: M = 1:0.8). Pruritus was the commonest symptom found in 25 (59.5%) patients followed by burning sensation (20, 47.6%), pain (4, 9.5%), oozing (3, 7.1%) and scaling (2, 4.8%). Thirty-four (80.9%) patients were exposed to more than one color. Red color was the commonest (30, 71.4%) color used, followed by green (17, 40.5%), blue (7, 16.7%), black (7, 16.7%), pink (6, 14.3%), silver (5, 11.9%), violet (4, 9.5%), and golden yellow (4, 9.5%). Cutaneous involvements were limited to the sites of application of colors. Among different types of cutaneous affections [Table 1], eczematous lesions were the most commonly found pattern [Figure 1], present in 24 (57.1%) patients. Other patterns exhibited were erosions in 9 (21.4%), xerosis and scaling in 7 (16.7%), erythema in 7 (16.7%), urticaria in 4 (9.5%), acute nail fold inflammation in 3 (7.1%) and abrasions in 3 (7.1%) patients. Face was the most common site affected [Figure 2] involving 24 (57.1%) patients followed by dorsum of hands (14, 33.3%), scalp (12, 28.6%), forearm (9, 21.4%), palms (8, 19%), arms (8, 19%) and trunk (3, 7.1%). Preexisting dermatoses were exacerbated in 13 (30.9%) patients including acne in 6 (14.3%), eczema in 6 (14.3%), and chronic paronychia in a single patient. The lesions were complicated with secondary pyoderma in 3 (7.1%) patients. Eight patients (19%) attributed hand involvement to activities related to preparation of colored solutions [Figure 3]. Eleven patients (26.2%) reported vigorous scrubbing with abrading materials to remove the color from their body. Three (7.1%) of these patients developed facial abrasions. Ocular complaints were seen in 7 (16.7%) patients including redness (3, 7.1%), watering (2, 4.8%), and grittiness (2, 4.8%).

**Table 1:** Cutaneous reaction patterns to *Holi* colors (n = 42)

Morphology	No	%*
Eczematous	24	57.1
Erosion	9	21.4
Xerosis and scaling	7	16.7
Erythema	7	16.7
Urticaria	4	9.5
Acute nail fold inflammation	3	7.1
Abrasion	3	7.1
Exacerbation of preexisting dermatoses	13	30.9

* The percentages add up to more than 100 as some patients had more than one patterns

## Discussion

Originally, a festival to celebrate good harvests and fertility of the land and to welcome spring with its new life and colors, *Holi* is now a symbolic commemoration of a legend from Hindu mythology which provides some of the ingredients for the celebrations.[1] The legend centers on an arrogant demon king who repeatedly tries but fails to kill his son Prahlada for worshipping Lord Vishnu. Finally, the king's sister Holika, who is blessed to be immune to burning, sits with the boy in a huge fire. However, while the prince Prahlada emerges unhurt with the blessings of the Lord, his aunt burns to death. Huge bonfires are burnt on the eve of *Holi* as a symbolic mark of this event from mythology. This vibrant festival is also associated with the legends of immortal love between Lord Krishna and Radha.

**Figure 4:**
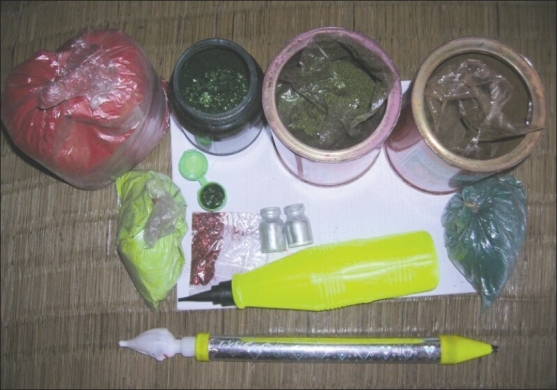
*Holi* colors and *pichkaris* (syringes for squirting color solutions)

Despite its religious roots, little religious activities are involved in the celebration and the central ritual of *Holi* involves throwing of colors in various forms on one another. The colors used during the Holi come in different forms including pastes, colored powders, and watercolors. Colors are often thrown at people with the help of long syringes called *pichkaris* [Figure 4] or balloons filled with watercolors, or simply smeared on the face. Some of the popular colors and their ingredients are black (lead oxide), green (copper sulfate and malachite green), silver (aluminum bromide), blue (Prussian blue), and red (mercury sulfate).[2] The dry colors, commonly known as ‘*gulals’* or ‘*abeer’*, have two components – a colorant and a base, both of which may cause cutaneous problems. Mica dust is often added as a sparkling agent to the dry powders that can lead to multiple microtraumas of skin and predisposition to infections.[3] Use of contaminated starch or wheat flour can further increase the chances of skin or ocular infections.

Although various ocular problems including conjunctivitis, corneal abrasions, and periorbital necrotizing fasciitis have been described to occur due to contact with *Holi* colors,[3–5] patterns of *Holi*-related cutaneous affections have hitherto been unreported in literature. Although definite cause-effect relationship was not established in our cross-sectional observational study, it has emerged that skin problems occur quite frequently following the celebration with colors. Most of the patients complained of pruritus and burning sensation and eczematous reaction pattern was found to be the commonest clinical findings in our patients (54.1%). There was no definite correlation between the hue of the color reported to be used and the cutaneous reaction patterns. The face was found to be the most commonly affected site presumably due to the fact that it was the usual site to which colors were applied. Hand involvement in some patients was attributed to immersion of hands in colored solutions during their preparation. In addition, different measures including vigorous scrubbing to remove the stains after the celebration also caused abrasions of skin. Thus, the dermatoses related to the celebration of Holi were presumably due to irritant or allergic contact dermatitis as well as mechanical factors. A limitation of our study has been our inability to perform appropriate patch tests to detect the precise etiology because the colorants were not available with the patients at the time of presentation and the reactions could have been due to unknown industrial dyes. A significant number of patients reported exacerbation of pre-existing dermatoses like acne vulgaris, eczema, and paronychia. Associated mild ocular involvements were also observed in some patients in the form of redness, watering, and grittiness.

*Holi* colors are produced in India without any quality checks and are sold freely in the market. Even when these are available in a packaged form, there is little information for the consumer about the source of the colors, their contents, and possible toxic effects. In recent years, several nongovernmental organizations have started campaigning for safe practices related to the use of colors. Some are producing and marketing ranges of safer colors derived from natural sources such as vegetables and flowers.[2] We believe that large-scale efforts to increase public awareness regarding the health hazards of harmful colors, widespread availability of safer alternatives at affordable prices, and governmental regulatory control on the production and selling of hazardous chemicals will go a long way in prevention of cutaneous and ocular diseases resulting from the celebration of this vibrant festival.
